# A novel dendritic mesoporous silica based sustained hydrogen sulfide donor for the alleviation of adjuvant-induced inflammation in rats

**DOI:** 10.1080/10717544.2021.1921075

**Published:** 2021-06-01

**Authors:** Yue Yu, Zhou Wang, Qinyan Yang, Qian Ding, Ran Wang, Zhaoyi Li, Yudong Fang, Junyi Liao, Wei Qi, Keyuan Chen, Meng Li, Yi Zhun Zhu

**Affiliations:** a State Key Laboratory of Quality Research in Chinese Medicine & School of Pharmacy, Macau University of Science and Technology, Taipa, China; b Shanghai Key Laboratory of Bioactive Small Molecules & School of Pharmacy, Fudan University, Shanghai, China

**Keywords:** S-propargyl-cysteine, dendritic mesoporous silica, endogenous hydrogen sulfide, sustained hydrogen sulfide donor, adjuvant-induced arthritis

## Abstract

**Purpose:**

S-propargyl-cysteine (SPRC), an excellent endogenous hydrogen sulfide (H_2_S) donor, could elevate H_2_S levels via the cystathionine γ-lyase **(**CSE)/H_2_S pathway both *in vitro* and *in vivo*. However, the immediate release of H_2_S *in vivo* and daily administration of SPRC potentially limited its clinical use.

**Methods:**

To solve the fore-mentioned problem, in this study, the dendritic mesoporous silica nanoparticles (DMSN) was firstly prepared, and a sustained H_2_S delivery system consisted of SPRC and DMSN (SPRC@DMSN) was then constructed. Their release profiles, both *in vitro* and *in vivo*, were investigated, and their therapeutical effect toward adjuvant-induced arthritis (AIA) rats was also studied.

**Results:**

The spherical morphology of DMSN could be observed under scanning Electron Microscope (SEM), and the transmission electron microscope (TEM) images showed a central-radiational pore channel structure of DMSN. DMSN showed excellent SPRC loading capacity and attaining a sustained releasing ability than SPRC both *in vitro* and *in vivo*, and the prolonged SPRC releasing could further promote the release of H_2_S in a sustained manner through CSE/H_2_S pathway both *in vitro* and *in vivo*. Importantly, the SPRC@DMSN showed promising anti-inflammation effect against AIA in rats was also observed.

**Conclusions:**

A sustained H_2_S releasing donor consisting of SPRC and DMSN was constructed in this study, and this sustained H_2_S releasing donor might be of good use for the treatment of AIA.

## Introduction

Rheumatoid arthritis (RA) is a chronic complex inflammatory disease, which might cause severe joint swelling, destruction and disability, (Smolen and Steiner 2003; Stamp and Cleland 2007; McInnes and Schett 2011) with subsequent high morbidity and mortality, and it is reported that RA affects about 1% of the world population (Rutherford et al. 2017). Although the cause of RA remains unclear for now, the immune mediators (inflammatory cytokines) is thought to induce the joint damage that begins at the synovial membrane (Low et al. 2008; Thomas et al. 2011; Davignon et al. 2013). Recently, various therapeutic agents have been applied for the treatment of RA, such as tofacitinib, methotrexate, monoclonal antibody and glucocorticoids (Chan et al. 2007; Bossaller and Rothe 2013; Lee et al. 2014; Lopez-Olivo et al. 2014; Ferreira et al. 2016). However, the toxicity adverse effects of these agents are unignorable, somehow limiting their clinical use (Chan et al. 2007).

For a long period, hydrogen sulfide (H_2_S) was considered as toxic compound. However, recently, H_2_S has been found to play an important function in physiological processes as the third endogenous gasotransmitter together with nitric oxide (NO) and carbon monoxide (CO). Moreover, the functions include neuromodulation, (Eto et al. 2002; Chen et al. 2007; Hu et al. 2010; Davoli et al. 2015) protection against cardiovascular system (Papapetropoulos et al. 2009; Mustafa et al. 2011; Bełtowski and Jamroz-Wis¨niewska 2014; Katsouda et al. 2016) and regulation of inflammation (Ishibashi 2013; Burguera et al. 2017).

Due to the significant contributions of H_2_S in many physiological processes, researchers often use H_2_S releasing agents (H_2_S donors) to modulate the H_2_S levels *in vitro* and *in vivo*. Commonly, sodium hydrosulfide (NaHS) and sodium sulfide (Na_2_S), as exogenous H_2_S donors, are mostly studied (Streng et al. 2008; Lu et al. 2010). Whereas, these exogenous H_2_S donor could release H_2_S upon addition to water in an uncontrolled and rapid manner, making it hard to precisely maintain H_2_S level both *in vitro* and *in vivo* (Lee, Zhou, et al. 2011; Lougiakis et al. 2016). To solve this problem, a series of endogenous H_2_S donor has been proposed, such as diallyl trisulfide (DATS), S-allyl cysteine (SAC), S-propargyl-cysteine (SPRC, also known as ZYZ-802). Our previous studies revealed that SPRC, an endogenous H_2_S modulator, exerted protective effects against inflammation by elevating the expression of cystathionine γ-lyase (CSE) (Wang et al. 2010; Liang et al. 2014). Recently, our group reported that SPRC could alleviate inflammatory symptoms in adjuvant-induced arthritis via Nrf2-ARE signaling pathway, which firstly reported the possibility of SPRC for the treatment of RA. However, when using high dosage of SPRC (for example, around 150 mg kg^−1^ according to rats’ body weight), its instant release of H_2_S *in vivo* might bring unacceptable side effects. Herein, how to attain a sustained and controllable H_2_S release remains a challenging problem for the further development of SPRC. Intended to solve the so-mentioned problems of SPRC, a series delivery system for SPRC has been established. However, the sustained promotion of *in vivo* H_2_S still remains a challenge (Huang et al. 2013; Tran et al. 2015, 2019a).

Nanoparticles have been widely used to conduct more specific and efficient treatments for complex diseases, (Kumari et al. 2010; Sahni et al. 2011; Mieszawska et al. 2013) thereinto, the conventional mesoporous silica nanoparticles (CMSN) has proved to be effective treatment for various diseases, (Balas et al. 2006; Li et al. 2015; Mccarthy et al. 2016) and not surprisingly, for inflammatory disease (Lee, Yun, et al. 2011; Braz et al. 2016). CMSN, with large surface area, pore volume, adjustable pore sizes, good biocompatibility, and easily modified pore surface, has become an ideal drug carrier. However, the clinical use of CMSN is limited partially due to its potential cellular toxicity. Herein, a novel silica-based nanocarriers with dendritic center-radial oriented mesopores (DMSN) was fabricated, (Shen, Yang, et al. 2014; Yang et al. 2015; Liu et al. 2018) and its use for loading of SPRC was investigated in this study.

Here in this passage, we hypothesized that DMSN could effectively load and sustained release SPRC *in vitro* and *in vivo*, further, attaining the sustained release of H_2_S. Adjuvant-induced arthritis (AIA) model in rats was successfully established to mimic RA in rats, and the use of SPRC@DMSN for the alleviation of AIA in rats was also studied. To the best of our knowledge, this is the first report about using DMSN as a delivery system on SPRC.

## Material and methods

### Chemical materials

SPRC was synthesized as we reported previously (Wang et al. 2009). Tetraethyl orthosilicate (TEOS) was purchased from Aladdin Reagent Co., Ltd (Shanghai, China). Cetyltrimethylammonium chloride (CTAC) solution (25 wt % in H_2_O), 3-aminopropyltriethoxysilane (APTES), triethanolamine (TEA) and decahydronaphthalene (DHA) were all purchased from Sigma Aldrich (St Louis, USA). Complete Freund’s Adjuvant (CFA), propargylglycine (PAG), BMDM medium (Iscove’s modified Dulbecco’s medium supplemented with 10% fetal calf serum, 100 μ mL^−1^ penicillin, 100 μg mL^−1^ streptomycin, 10 mM thioglycerol), human macrophage-colony stimulating factor (M-CSF), sodium hydroxide, hydrochloric acid, acetonitrile, methanol, toluene, anhydrous ethanol, sodium sulfide, monobromobimane (MBB), diethylenetriaminepentaacetic acid (DTPA), sulfosalicylic acid and hydrocortisone were purchased from Macklin Industrial Corporation (Shanghai, China). Elisa kit of TNF-α, IL-1β, IL-6 and IL-10 were purchased from MultiSciences (Hangzhou, China).

### Synthesis of dendritic mesoporous silica nanoparticle

The DMSN was prepared via one-pot synthesis as reported (Shen, Yang, et al. 2014) with little modification. 50 mL CTAC solution (25 wt%) and 0.4 g of TEA were added to 80 mL of deionized water with stirring at 60 °C for 1 h. 40 mL TEOS-DHA solution (20 v/v%) was then dropped into the mixture and the reaction was kept magnetic stirring for another 12 h in 60 °C water bath. The products were centrifugated, and the precipitation was washed with ethanol for several times and calcined at 550 °C to remove the template and residual reactants. FITC-conjugation was done by adding FITC in the synthesis step to create inherently fluorescent DMSN as reported (Huang, Huang, et al. 2011; Huang, Li, et al. 2011; Luo et al. 2011; Desai et al. 2016).

### Physicochemical characterization

The particle size and ζ potentials of the nanoparticles were measured by Malvern Zetasizer Nano Series (Malvern, USA). The SEM (Nova Nano SEM, Philips, Netherlands) and TEM (JEM2100F High Resolution JEOL, Japan) were used to observe the morphology and pore structure. The nitrogen adsorption-desorption was performed using a Micromeritics Tristar 3000 pore analyzer (Micromeritics, USA) to determine the pore size, pore volume, and specific surface area. Surface area was determined using the Brunauere Emmere Teller (BET) model, and the pore size distributions was calculated from the adsorption branch of the isotherm using the Barrette Joynere Halenda (BJH) model.

### SPRC loading into dendritic mesoporous silica nanoparticles

500 mg of prepared DMSN was suspended in different volume (5–20 mL) of SPRC saturated solution (50 mg mL^−1^), and the mixture kept magnetic stirring for 24 h at 25 °C. The resulting suspensions were centrifugated and the precipitates were collected, washed with distilled water for 3 times. The loading efficiency (LE) and encapsulation efficiency (EE) were determined using Agilent 1200 series HPLC system (Agilent Technologies, Palo Alto, CA). The SPRC loaded DMSN were named as SPRC@DMSN. Each sample was conducted in triplicate, and calculated as the following equation:

$$\mathrm{LE}\;(\%)\;=\;\frac{\text{weight of SPRC initially fed}-\text{weight of SPRC in supernatent}}{\text{weight of SPRC}@\mathrm{DMSN}}\mathrm{EE}\;(\%)\;=\frac{\text{weight of SPRC initially fed}-\text{weight of SPRC in supernatent}}{\text{weight of SPRC initially fed}}$$

### *In vitro* SPRC release study

To study the *in vitro* releasing ability, 20 mg of SPRC powder and SPRC@DMSN were firstly dispersed in 3 mL of phosphate buffer saline (PBS, pH = 7.4) and then transferred into a dialysis bag (cut off molecular weight 12–14 kDa), which were sealed and immersed in 47 mL of PBS at 37 °C under stirring. At predetermined time intervals, 1 mL of sample solution was taken out and immediately replenished with an equal volume of PBS. The sample solution was centrifugated (10,000 rpm, 10 min) and further analyzed by HPLC.

### Macrophages culture

Bone marrow derived monocytes were isolated from healthy mice. Mice were firstly sacrificed and after cut the epiphyses of the bones, further flushing the marrow into a 50 mL centrifuge tube using a 5 mL syringe and a 23 G needle. The cell suspension was then filtered and centrifuged. The supernatant was then discarded and resuspend the cell pellet in 3 mL of BMDM medium. Dilute the cells at a concentration of 3.5 × 10^5^ cells mL^−1^, then added M-CSF at a final concentration of 25 ng mL^−1^ and seeded 10 mL per 10 cm cell culture-treated petri dish. Finally, incubated at 37 °C with 5% CO_2_ for 3 days, and replaced the medium and incubated for another 3 days (Assouvie et al. 2018).

### Cytotoxicity toward macrophages

Macrophages were seeded into 96-well plates at a density of 1 × 10^4^ per well and incubated in 5% CO_2_ atmosphere at 37 °C for 24 h. The original medium was then replaced with 200 μL of solutions containing various concentrations of SPRC, and SPRC@DMSN for 48 h incubation. The numbers of living cells were determined using the CCK-8 assay as reported.(Zheng et al. 2012) To verify the cellular uptake of DMSN by macrophages, cells were seeded in confocal microscopy 20 mm^2^ Petri dish for 12 h, then added the FITC-labeled DMSN (10 mg mL^−1^) to medium and co-incubated for 1, 6, and 12 h followed by PBS washing for three times. The cell nucleus was stained using DAPI.

### *In vitro* H_2_S release study

Samples of macrophages were transfected with SPRC and SPRC@DMSN (equivalent to 10 μM SPRC) for 48 h. Supernatant was collected at predetermined intervals for the detection of H_2_S release.

### Anti-inflammation *in vitro*

To investigate whether transfection with supplementations could inhibit the inflammation, LPS-stimulated macrophages were transfected with SPRC and SPRC@DMSN (equivalent to 10 μM SPRC) for 6 h. To prove the anti-inflammation effect was through the release of endogenous H_2_S, PAG (2 mM) was added to co-incubate with SPRC or SPRC@DMSN.

### Animal experiment design

The Animal Care and Use Committee of Municipal Affairs Bureau of Macau approved all studies described herein (approval number AL010/DICV/SIS/2018), and experiment was conducted under the guidance of NIH Guide for the Care and Use of Laboratory Animals (8th edition).

### Pharmacokinetics study

Samples of SPRC powder and SPRC@DMSN were dissolved or dispersed in saline for oral administration, each sample contains same amount of SPRC, and dosage were calculated through the weight of rats (100 mg kg^−1^). The rats’ serum was collected at predetermined time (0, 0.5, 1, 1.5, 2, 3, 6, 12, 24, 48, and 72 h) into heparin sodium tubes and analyzed.

### Alleviation of inflammation in rats

The AIA rat model was established via a subcutaneous injection of 100 μL of CFA (10 mg mL^−1^) at the base of the tail, while control group received 100 μL of saline. Totally 24 of rats were randomly divided into four groups as follows: *Control group* (*n* = 6), no intervention; *AIA group* (*n* = 6), injection of 100 μL of CFA; *SPRC group* (*n* = 6), after injection of 100 μL of CFA, further oral administrated with 2 mL of SPRC solution every 3 days (equivalent to 100 mg kg^−1^ of SPRC) for 30 days; *SPRC@DMSN group* (*n* = 6), after injection of 100 μL of CFA, further oral administrated with 2 mL of SPRC@DMSN suspension every 3 days (equivalent to 100 mg kg^−1^ of SPRC) for 30 days.

The paw volume was measured by a ugo basile 7140 plethysmometer (Ugo Basile, Gemonio VA, Italy) and body weight was measured at the 0, 5th, 15th, 20th, 25th, and 30th day post the injection of CFA. Arthritis index was scored from 0 to 4 per limb, for 0 = no sign of inflammation, 1–4 = increasing degrees of inflammatory, with a maximum score of 16 per rat (Chuang et al. 2019).

At day 30, blood sample was collected from rats in each group, the pro-inflammatory cytokines (TNF-α, IL-1β and IL-6) and anti-inflammatory cytokine (IL-10) levels in serum were measured using ELISA kits according to the manufacturer’s instructions.

### Histopathological analysis

Safranin-O staining, which stains proteoglycan in cartilage, was carried out by previously described methods (Ray et al. 2004; Chen et al. 2020; Shin et al. 2020). In brief, dewaxed tissue sections were stained with Weigert’s hematoxylin working solution composed of 1% hematoxylin and 30% ferric chloride (anhydrous) for 10 min. After extensive washing, sections were counter-stained with fast green solution for 5 min. After rinsing with 1% acetic acid, sections were then stained with 0.1% safranin-O solution for another 5 min.

### Micro-CT analyses

The hind limb of each rat was harvested after sacrifice and imaged with 3 D microcomputed tomography (micro-CT, Siemens Inveon MM Gantry CT, Germany) at a voltage of 70 kV and an electric current of 400 µA. The exposure time was 800 ms, and the scan area was 26.42 mm × 26.42 mm × 30 mm around the metatarsal bone articulations.

### Safety evaluations

The white blood cell (WBC), red blood cell (RBC), and hemoglobin (HGB) were assayed using standard methods (Sysmex KX-21, Japan). Major organs including heart, liver, spleen, lung, and kidney were dissected and fixed with 4% paraformaldehyde and further stained with H&E.

### Measurement of H_2_S concentration

Concentration of H_2_S was measured as reported with little modification (Zhu et al. 2007). Briefly, 15 μL serum or culture medium, 25 μL MBB acetonitrile solution, and 35 μL 0.3% diethylenetriaminepentaacetic acid (DTPA) containing Tris-HCl buffer (pH 9.5) were mixed and incubated in hypoxia incubator for 30 min. Subsequently, 25 μL sulfosalicylic acid was added to stop reaction and then centrifugated at 12,000 rpm for 10 min. Finally, 30 μL supernatant, 267 μL acetonitrile and 3 μL internal standard (hydrocortisone methanol solution) were mixed and analyzed with LC-MS.

Samples were analyzed using an Agilent 1200 series HPLC system (Agilent Technologies, Palo Alto, CA) coupled with an Agilent 6460 Triple Quadrupole (Agilent Technologies, Palo Alto, CA). ZORBAX Eclipse Plus 95 C18, 2.1 × 50 mm, 1.8 μm column was used and temperature was set at 35 °C. The mobile phase consisted of water (A) and acetonitrile (B) and was delivered gradient at 0–0.5 min, 5% B; 0.5–0.6 min, 5–20% B; 0.6–5.0 min, 20–47.5% B; 5.0–5.1 min, 47.5–95% B; 5.1–6.0 min, 95% B, at a flow rate of 0.3 mL min^−1^. The mass spectrometer was operated in positive ion mode. Scan type was chosen MRM with gas temperature at 325 °C and gas flow at 10 L min^−1^. Scan time was 500 ms and start-stop mass was 100–1000. The sample injection volume was 5 μL.

### Western blot analysis

Western blot analysis was performed as previously described (Pan et al. 2011). 50 μg of proteins from cell or tissue were separated and transferred to polyvinyl difluoride membrane, with further blocking with 5% nonfat dried milk. The membranes were then probed with antibodies against CSE and GAPDH and incubated with either horseradish peroxidase-conjugated goat anti-rabbit or anti-mouse antibody. Immunoreactive proteins were visualized by enhanced chemiluminescence and signal intensity was detected and quantified by Alpha Imager (San Leandro, USA).

### Statistical analysis

Each experiment was performed at least triplice and the data are presented as the mean ± SD. Statistical significance was determined using one-way analysis of variance (ANOVA) test and *p* < 0.05 was considered significant unless otherwise indicated.

## Results

### Characterization of SPRC@DMSN

**Figure 1.**
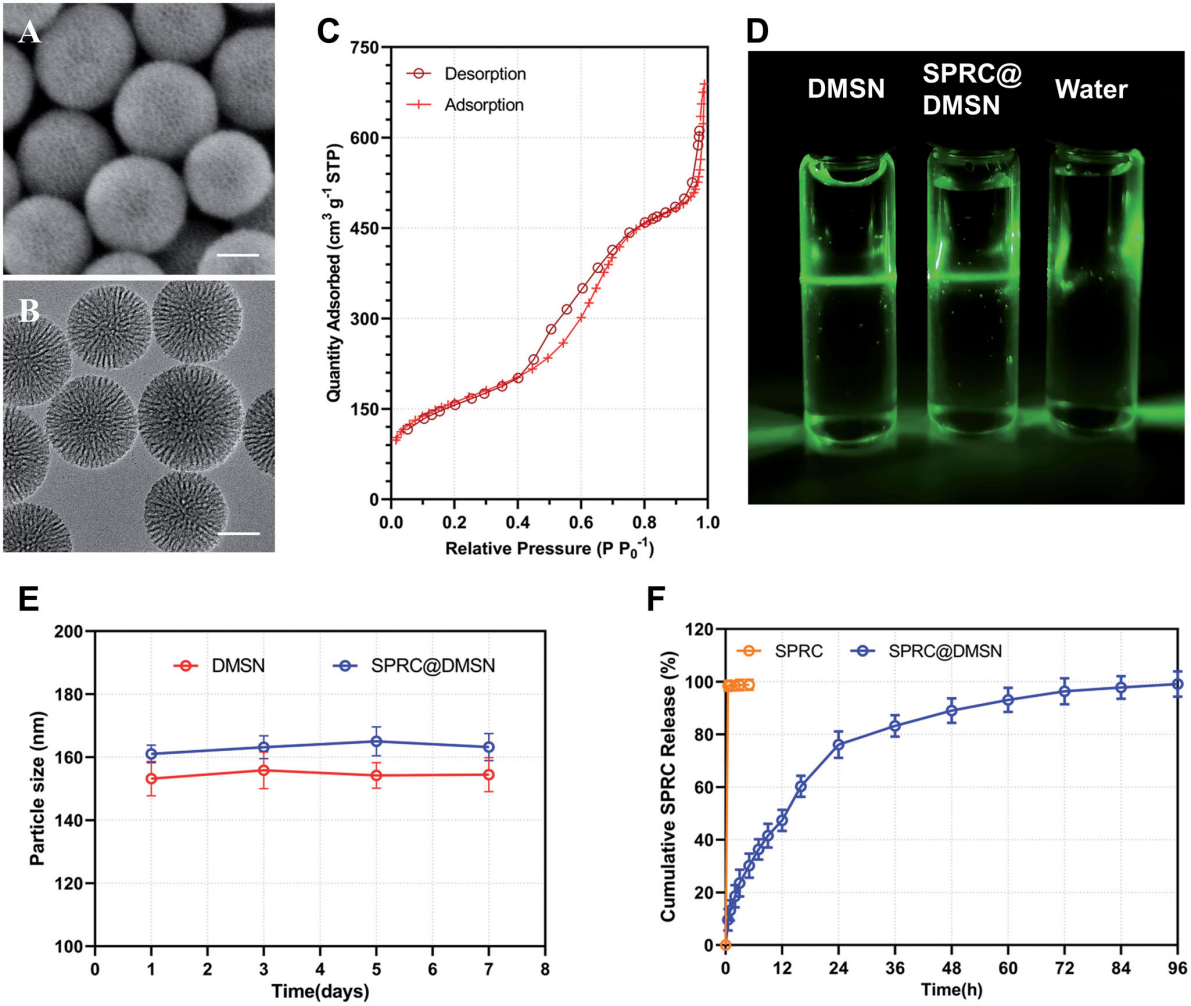
The morphology study of prepared DMSN and SPRC@DMSN. The SEM of (A) and TEM of (B) DMSN (Scale bar = 50 nm). (C) The typical type-IV isotherm containing H1-type hysteresis indicated the preparation of mesopore structure in DMSN. (D) The Tyndall effect could be observed when DMSN and SPRC@DMSN dispersed in water and (E) the 7-day stability of DMSN and SPRC@DMSN in water. (F) The cumulative release of SPRC in PBS (pH = 7.4) within 96 h (*n* = 3, mean ± SD).

The spherical morphology of DMSN could be observed under SEM (Figure 1(A)), while the TEM images showed the central-radiational pore channel structure of the prepared DMSN (Figure 1(B)). According to the N_2_ adsorption − desorption isotherms, the prepared DMSN (Figure 1(C)) exhibited a typical type-IV isotherm containing H1-type hysteresis, suggesting the mesoporous structure. Meanwhile, a capillary condensation step around 0.2 < P P_0_^−1^ < 0.4 could be observed, which indicated a clear evidence for their narrow pore size. The influence of different amounts of SPRC used on the preparation of SPRC@DMSN was investigated, and the results of particle size, ζ potential, pore size, pore volumes, surface area, loading efficiency (LE), and encapsulation efficiency (EE) of the prepared DMSN and different SPRC@DMSN were listed in Table 1. Interestingly, the diameter of DMSN was around 100 nm calculated under TEM while the hydrodynamic diameter for DLS test showed a particle size around 150 nm (Table 1), which might be resulted from the aggregate property of nanoparticles in water. (van Grieken et al. 2009). Both the pore volume and surface area showed a decreasing tendency before 15 mL of SPRC saturated solution used, indicated a successfully loading of SPRC into DMSN. Though an increase tendency of LE could be observed when the SPRC saturated solution volume increased, a ‘loading capacity peak’ would be observed when the SPRC saturated solution volume went beyond 15 mL, hence above all, the volume was chosen 15 mL for further study, and for a convenient expression, the SPRC@DMSN (15 mL) was named as SPRC@DMSN. The Tyndall effect could be observed in Figure 1(D). Moreover, both DMSN and SPRC@DMSN exhibited excellent *in vitro* stability, with negligible changes of particle size during storage under room temperature (25 °C) for a week (Figure 1(E)). In the *in vitro* SPRC releasing experiment (Figure 1(F)), the SPRC powder showed immediately and complete dissolve within few minutes, while SPRC@DMSN showed prolonged and sustained SPRC release within 96 h, which indicated a sustained releasing ability of SPRC@DMSN.

**Table 1.** The characterization of DMSN and SPRC@DMSN (*n* = 3, mean ± SD).

Parameters	DMSN	SPRC@DMSN (5 mL)	SPRC@DMSN (10 mL)	SPRC@DMSN (15 mL)	SPRC@DMSN (20 mL)
PS (nm)	154.3 ± 4.5	158.0 ± 3.8	158.9 ± 4.0	160.3 ± 3.9	161.8 ± 4.3
ZP (mV)	−20.2 ± 1.7	−20.4 ± 1.5	−21.1 ± 1.6	−21.4 ± 1.2	−21.5 ± 1.5
PoS (nm)	3.09 ± 0.04	3.10 ± 0.05	3.09 ± 0.03	3.09 ± 0.04	3.10 ± 0.05
PV (cm^3^ g^−1^)	1.07 ± 0.05	0.83 ± 0.04	0.61 ± 0.06	0.33 ± 0.04	0.31 ± 0.04
SA (m^2^ g^−1^)	577.78 ± 2.56	432.39 ± 3.15	327.13 ± 2.24	169.17 ± 2.85	170.48 ± 3.06
LE (%)	–	14.44 ± 0.77	21.06 ± 0.93	23.62 ± 0.81	23.43 ± 0.97
EE (%)	–	33.75 ± 2.12	26.69 ± 1.49	20.63 ± 0.92	15.30 ± 0.83

*Note*. PS: particle size; ZP: ζ potential; PoS: pore size; PV: pore volume; SA: surface area; LE: loading efficiency; EE: encapsulation efficiency.

### Cytotoxicity study

**Figure 2.**
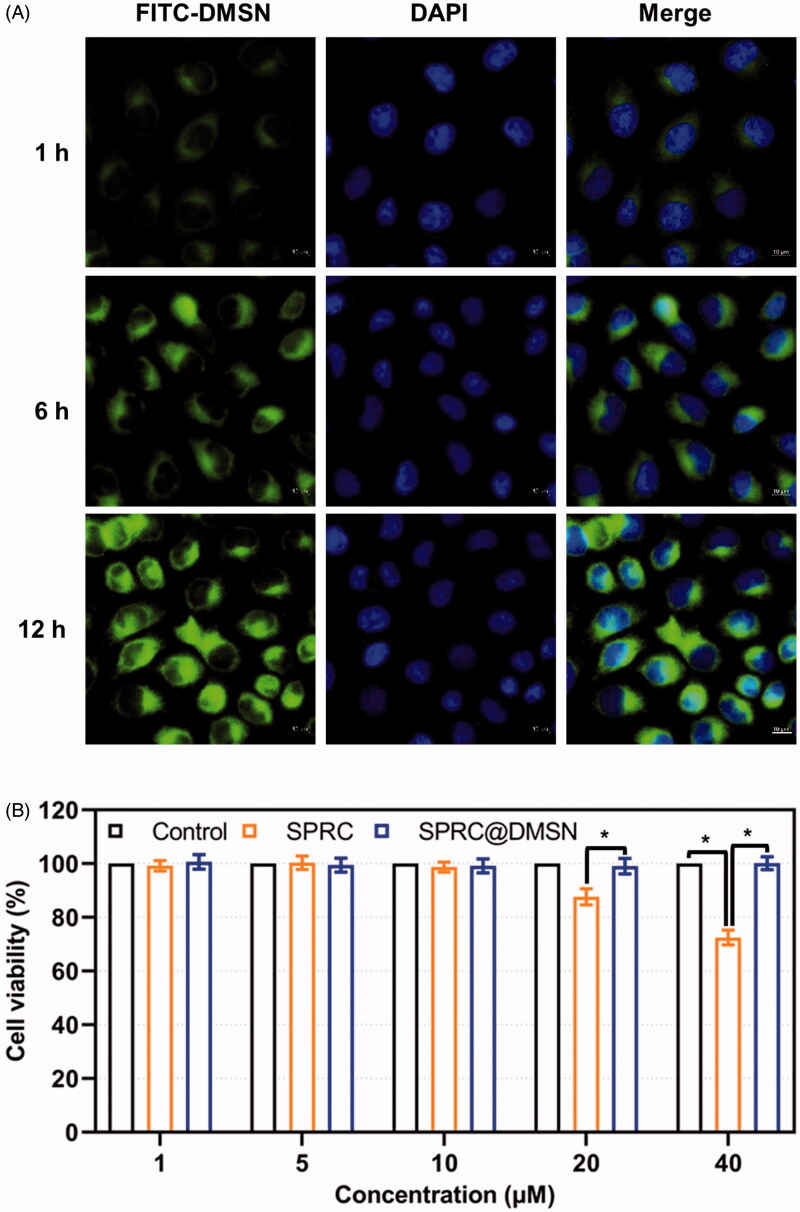
(A) The time-dependent cellular uptake of FITC labeled DMSN (Scale bar = 10 μm). (B) Cell viability after exposure to different concentration of SPRC and SPRC@DMSN. Significant different compared with corresponding group indicated as (*) (*n* = 3, mean ± SD).

A time-depend high green fluorescence could be observed in macrophages, which indicated the cellular uptake of the FITC labeled DMSN (Figure 2(A)), and cell viability remained around 95% or higher when the concentration of SPRC changed from 1 to 10 μM in SPRC or SPRC@DMSN groups (Figure 2(B)). However, the SPRC group showed a decrease of cell viability at concentration of 20 and 40 μM. Interestingly, the SPRC@DMSN group showed negligible cytotoxicity at even high SPRC concentration (equal to 20 and 40 μM SPRC), which revealed the lower cytotoxicity of SPRC@DMSN group.

### Supplementations exerted anti-inflammation effects through elevation of H_2_S level via CSE/H_2_S pathway *in vitro*

**Figure 3.**
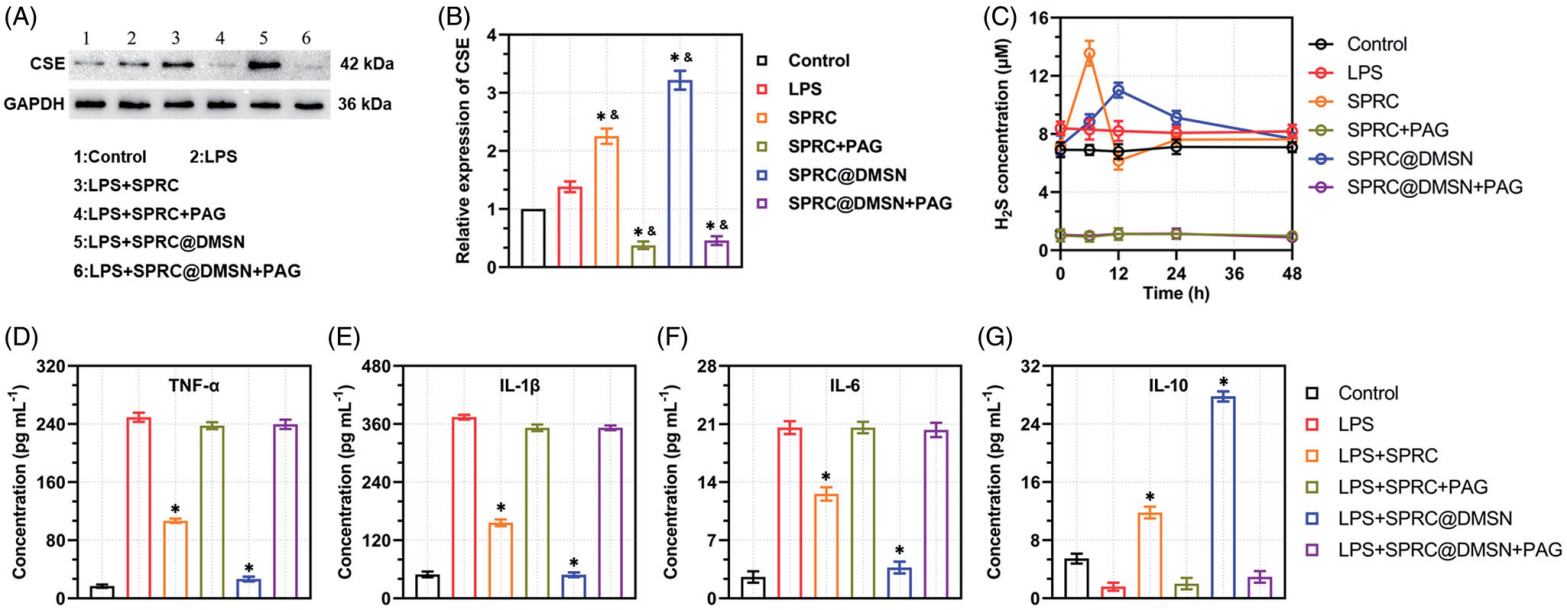
Supplementations exerted anti-inflammation effects via CSE/H_2_S pathway *in vitro*. (A, B) Changes of CSE expression after co-incubation with different supplementations. Significant different compared with control group indicated as (*), and significant different compared with LPS group indicated as (&) (*n* = 3, mean ± SD). (C) The H_2_S release *in vitro* after co-incubation with different supplementations (*n* = 3, mean ± SD). The pro-inflammatory cytokines levels of (D) TNF-α, (E) IL-1β, (F) IL-6, and anti-inflammatory cytokine level of (G) IL-10 were measured. Significant different compared with LPS treated group indicated as (*) (*n* = 3, mean ± SD).

CSE was an important endogenous H_2_S producing enzyme, and SPRC was reported to elevate the H_2_S concentration via CSE/H_2_S pathway. A significant increase of expression of CSE could be observed in groups treated with SPRC or SPRC@DMSN, while the latter showed a stronger elevating effect. PAG, a CSE inhibitor, was then co-incubated. Not surprisingly, all group showed significant inhibition of CSE expression (Figure 3(A,B)).

To further prove the change of CSE expression could influence the release of H_2_S, we then detected the H_2_S release within 48 h. The control group showed stable H_2_S concentration around 7.22 μM, while treated with LPS could slightly increase the H_2_S level to around 8.31 μM as shown in Figure 3(C). Meanwhile, co-incubation with SPRC and SPRC@DMSN could significantly further increase the H_2_S level, while treated with SPRC showed an instant increase and decrease of H_2_S, and SPRC@DMSN group showed sustained release manner of H_2_S. However, the release of H_2_S was hardly observed in any group co-treated with PAG, which further proved that SPRC release H_2_S via CSE/H_2_S pathway.

As reported, macrophages could promote the release pro-inflammatory cytokines (such as TNF-α, IL-1β and IL-6) during the progression of AIA, while the release of anti-inflammatory cytokines (such as IL-10) was inhibited, and this abnormal homeostasis of cytokines might further initiate and perpetuate inflammation and bone destructive in the joint (Kinne et al. 2007; Szekanecz and Koch 2007; Udalova et al. 2016). As shown in Figure 3(D–G), 10 μM of SPRC suppressed the expression of LPS-induced pro-inflammatory cytokines (TNF-α, IL-1β and IL-6) release and increased the release of IL-10 detected by ELISA. Similarly, the SPRC@DMSN showed similar trend while the SPRC@DMSN showed better anti-inflammatory effect. However, their anti-inflammatory effects were all inhibited after co-incubation with PAG, the CSE inhibitor.

### Supplementation facilitated the production of endogenous H_2_S *in vivo*

**Figure 4.**
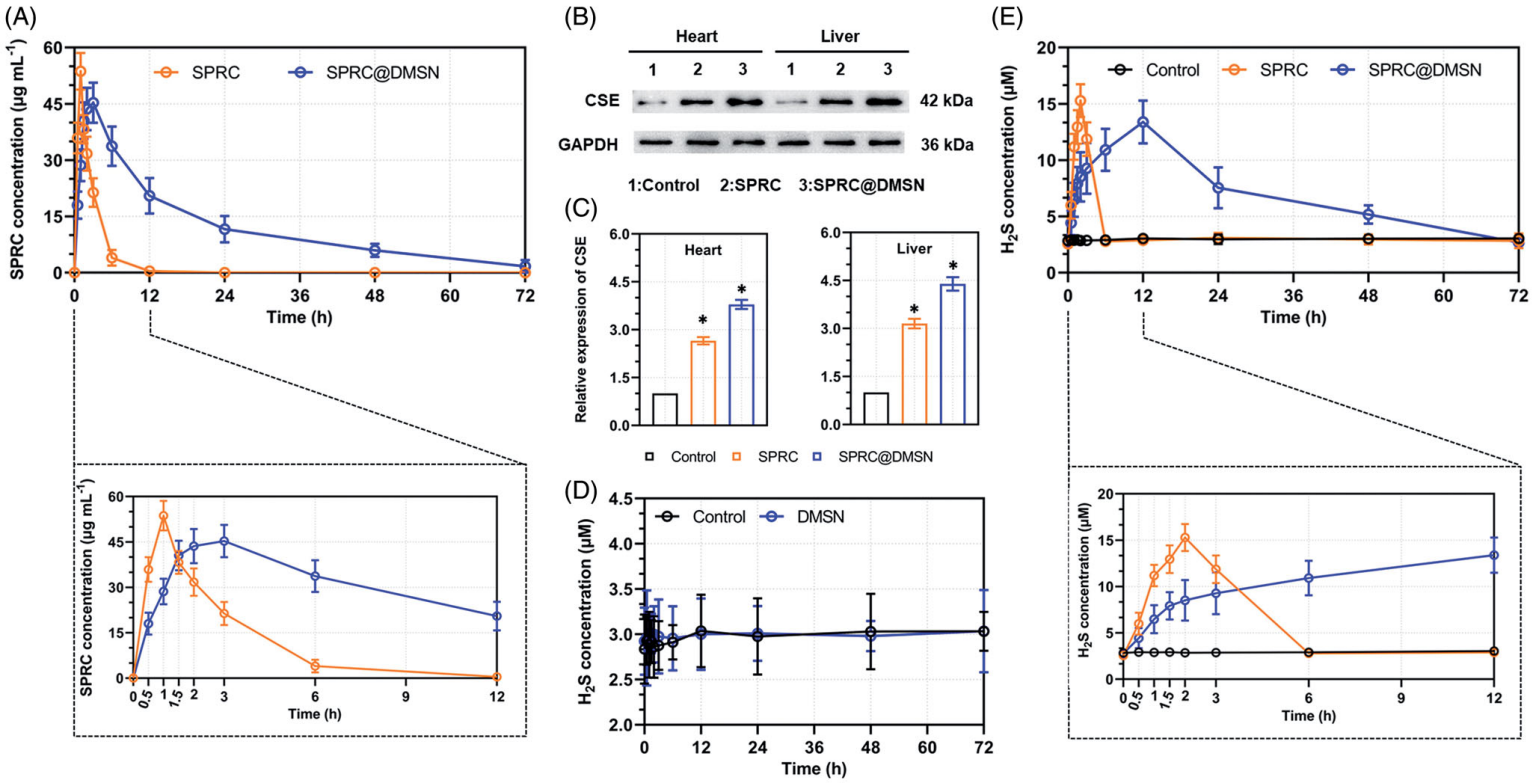
The supplementations promoted endogenous H_2_S release in plasma through CSE/H_2_S pathway after single administration. (A) The plasma concentration of SPRC within 72 h (above) and first 12 h (below). (B, C) Supplementations could elevate the CSE expression in heart and liver. (D) Administration of DMSN did not influence the H_2_S level *in vivo*, but (E) the administration of SPRC or SPRC@DMSN could elevated the plasma H_2_S concentration within 72 h (image of the first 12-h H_2_S plasma concentration was shown at bottom). SPRC or SPRC@DMSN were single oral administrated at dosage of 100 mg kg^−1^ according to rats’ body weight (*n* = 6, mean ± SD).

The 3-day SPRC plasma concentration profile after single administration of supplementations was shown in Figure 4(A), and the pharmaceutical parameters were listed in Table 2. The *C*_MAX_ of SPRC@DMSN (∼43.61 μg mL^−1^) was slightly lower than the SPRC (53.66 μg mL^−1^) though, the SPRC@DMSN group (17.09 h) showed dramatically prolonged *t*_1/2_ than the SPRC group (1.56 h), while meantime, the *T*_MAX_ was also increased from 1 h (SPRC group) to 3 h (SPRC@DMSN group). Notably, the SPRC@DMSN showed higher AUC than the SPRC.

**Table 2.** Pharmacokinetic parameters of SPRC and SPRC@DMSN (*n* = 6, mean ± SD).

Parameter	Unit	SPRC	SPRC@DMSN
*t*_1/2_	h	1.56 ± 0.23	17.09 ± 2.79
Tmax	h	1.00 ± 0.00	3.00 ± 1.00
Cmax	μg mL^−1^	53.66 ± 5.92	43.61 ± 4.75
MRT 0 − inf_obs	h	2.50 ± 0.36	21.95 ± 2.51
AUC 0 − t	μg ml^−1^ h^−1^	149.61 ± 17.82	789.14 ± 60.20
AUC 0 − inf_obs	μg ml^−1^ h^−1^	150.53 ± 22.58	824.70 ± 67.45

While SPRC was reported to promote H_2_S release *in vivo* through increasing the expression of CSE, which reported to be a main H_2_S releasing enzyme widely distributed in heart and liver, (Xin et al. 2016; Wang et al. 2017; Tran et al. 2019b) and oral administration of SPRC was found to increase the SPRC plasma concentration in an instant manner while the SPRC@DMSN in a sustained manner as illustrated above. The expression of CSE in liver and heart was firstly investigated, and results revealed that both SPRC and SPRC@DMSN could increase the expression of CSE (Figure 4(B–C)), while the SPRC@DMSN showed higher expression potentially due to its sustained releasing manner.

We then compared the H_2_S levels in plasma after single administration of SPRC, SPRC@DMSN, together with DMSN without SPRC loading. As shown in Figure 4(D), the rats in control group or group treated with DMSN showed constant concentration of H_2_S in plasma around 3 μM within 72 h, which indicated the stable plasma H_2_S concentration *in vivo*. After administration with SPRC, the plasma H_2_S concentration showed a significant instant increase and peaked at the 2-hour post administration (Figure 4(E)). Different from the immediately release of H_2_S promoted by of SPRC, the H_2_S production from SPRC@DMSN was relative slow compared with the SPRC group, which was potentially due to the sustained release manner of SPRC from SPRC@DMSN.

### Therapeutic efficacy of supplementation toward AIA in rats

**Figure 5.**
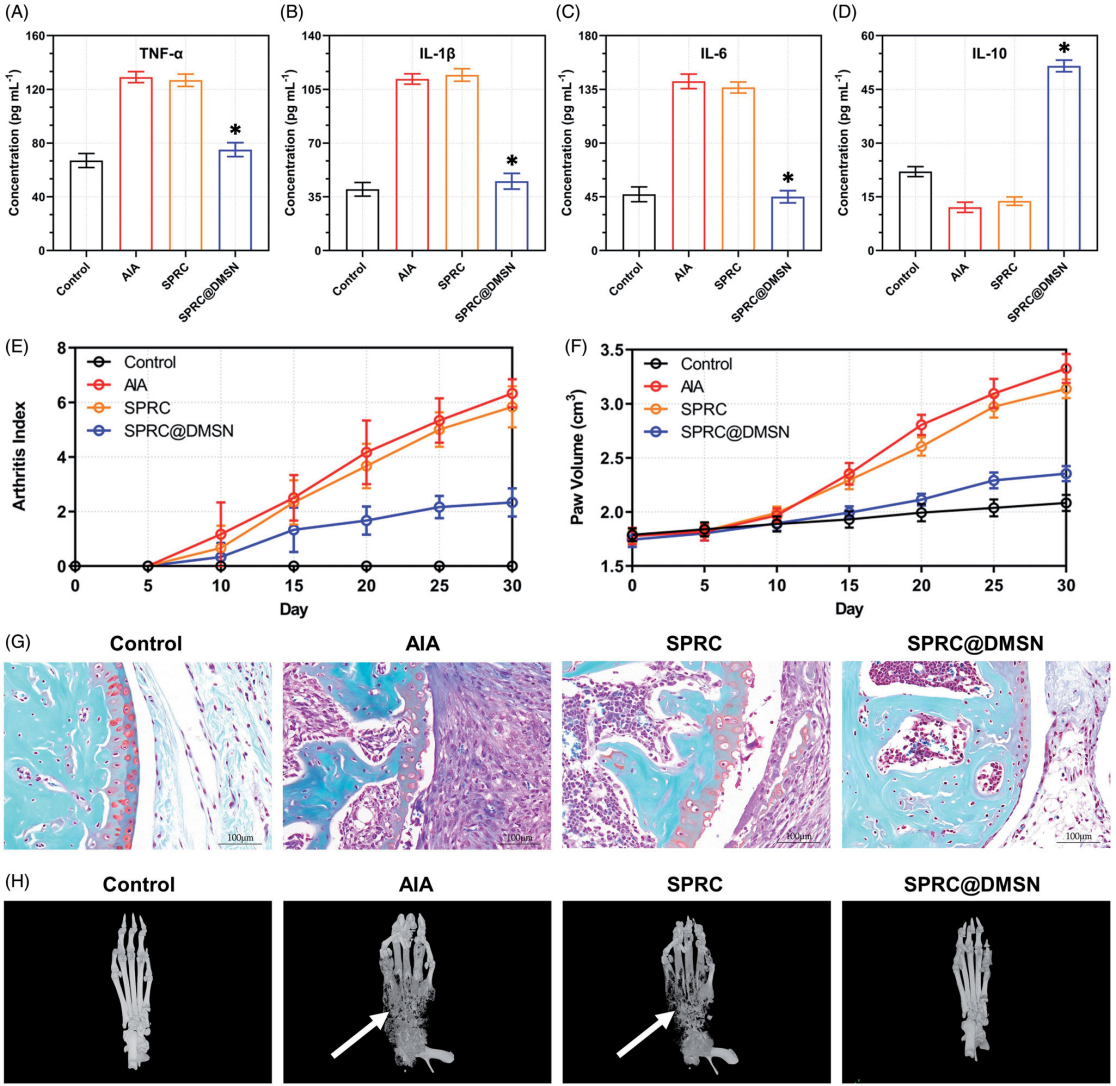
Supplementations mitigated AIA symptoms. The pro-inflammatory cytokines levels of (A) TNF-α, (B) IL-1β, and (C) IL-6 and anti-inflammatory cytokine level of (D) IL-10 in rats were measured. Significant different compared with AIA group indicated as (*) (*n* = 6, mean ± SD). (E) The arthritis index and (F) paw volume were used to evaluate the severity of swollen symptoms (*n* = 6, mean ± SD). (G) Safranin-O staining examination was conducted to evaluate the articular cartilage destruction. (Scale bar = 100 μm). (H) The micro 3 D analysis images of rats’ paws, 3 D reconstructions of paws from rats used the Mimics software. The erosions sites of bones indicated by the white arrow.

As a long-term autoimmune disorder, the pro-inflammatory cytokines (TNF-α, IL-1β, and IL-6) and anti-inflammatory cytokine (IL-10) were important parameters to assess the therapeutic effects in AIA rats. As shown in Figure 5(A–D), SPRC showed negligible decrease of pro-inflammatory cytokines and increase of anti-inflammatory cytokine. Meantime, the SPRC@DMSN showed significant anti-inflammatory effect. The alleviation of inflammation in AIA rats was studied through the paw volume and arthritis index (Figure 5(E,F)). From the results, no dramatic increase of paw volume and arthritis index in all groups before 10th day, while starting from 10th day, the AIA group and SPRC group showed a significant increase tendency, which indicated low therapeutic effect of SPRC. However, in SPRC@DMSN group, decrease level of paw volume and arthritis index could be observed. The SPRC@DMSN-treated rats had a marked reduction in cartilage degradation (Figure 5(G)) and bone erosion (Figure 5(H)) in comparison with the AIA group or SPRC-treated group.

### Safety evaluations

**Figure 6.**
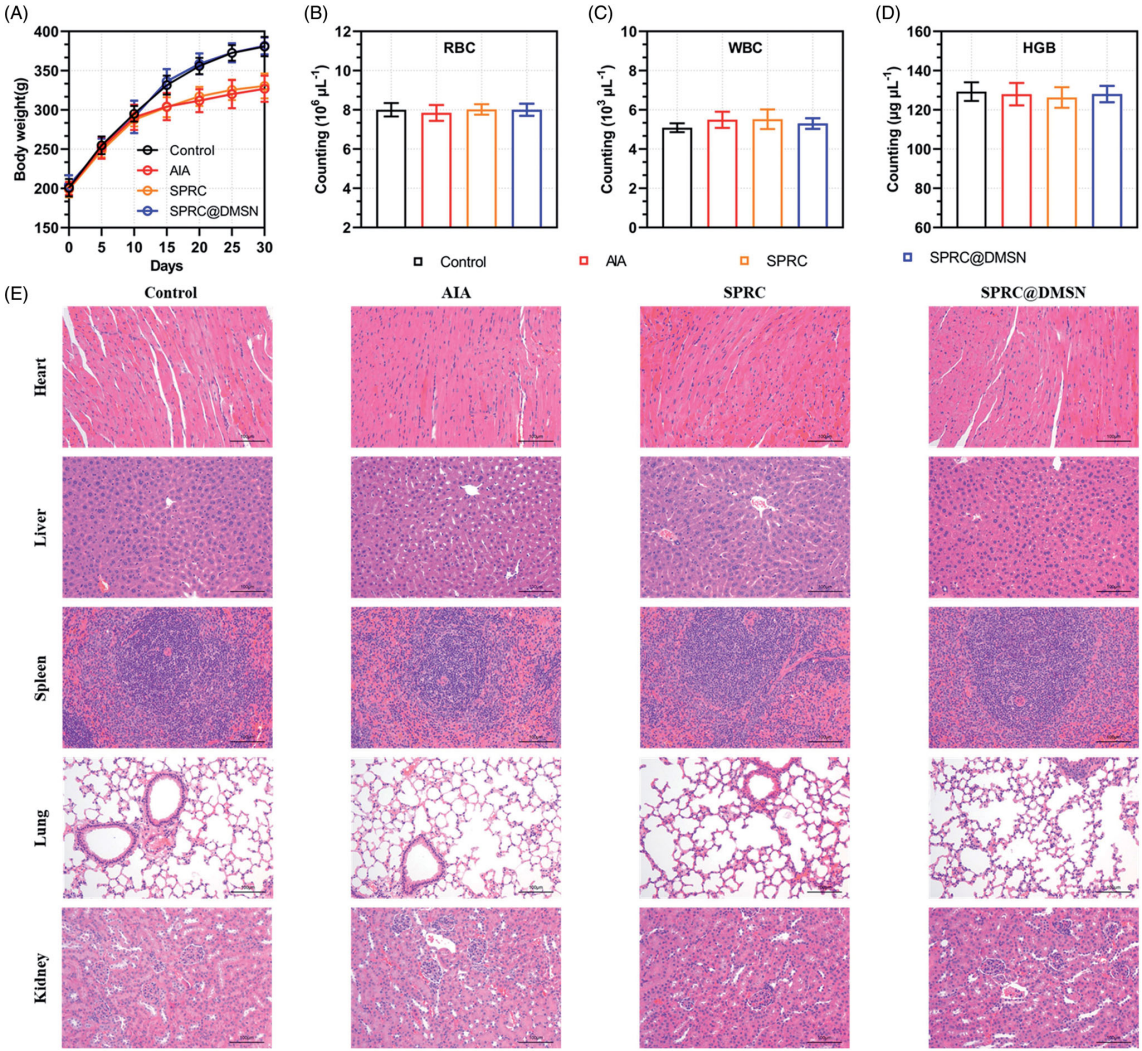
Evaluation of potential adverse effects of supplementations in AIA rats. (A) The body weight of rats was recorded every 5 days. After 30-day experiment, rats were sacrificed and (B) red blood cell (RBC), (C) white blood cell (WBC) count and (D) hemoglobin (HGB) were measured. (*n* = 6, mean ± SD). (E) H&E staining was carried out for the examination of heart, liver, spleen, lung, and kidney after 30 days’ experiment. Images were acquired at 200 × magnification (Scale bar = 100 μm).

Chronic administration of high doses of SPRC generally leads to side effects such as body weight loss and hematologic abnormalities, which might limit the use of SPRC (Zheng et al. 2012). During the progression of arthritis, the body weight changes of AIA rats presented in Figure 6(A). Compared with the control group, all other groups showed a decrease tendency in body weight might be due to the strong impact of AIA inflammation, especially starting from 10th day after induction of AIA model. At day 30, the body weight of control group (380.7 ± 12.32 g) was about 53 g heavier than the AIA group (327.1 ± 16.80 g) and 50 heavier than the SPRC group (330.6 ± 16.07 g), while the SPRC@DMSN (381.5 ± 10.86 g) showed a dramatic up-regulated of body weight loss at 30th day possibly because of the alleviation of inflammation symptoms. It was reported that a long-term exposure to even acceptable concentrations of H_2_S at workplace is a potential risk for human health (Saeedi et al. 2015), the hematologic study was conducted to evaluate the side effects of supplementations on rats, and results were shown in Figure 6(B–D). Except the white blood cell (WBC) count showed a slight increase in the AIA and SPRC groups compared with other groups, no significant difference observed in any other groups’ hematologic studies, neither in red blood cell (RBC) count nor the hemoglobin (HGB) levels. In addition, to evaluate possible side effects of supplementations, the heart, liver, spleen, lung, and kidney were resected from rats and examined by H&E staining. Compared with the control group, no tissue damage could be observed in any experimental groups, which implied the low toxicity of supplementations *in vivo* (Figure 6(E)).

## Discussions

SPRC, an excellent hydrophilic endogenous H_2_S donor, was reported to significantly mitigate the symptoms in diseases such as acute myocardial ischemia, (Wang et al. 2009) acute pancreatitis, (Tamizhselvi et al. 2010; Sidhapuriwala et al. 2012) and Alzheimer’s disease (Gong et al. 2011). However, SPRC might promote the release of H_2_S in an instant manner which could potentially cause neuronal toxicity with high dosage, thus limiting its further clinical use. Intended to solve the above problem, SPRC was successfully absorbed into DMSN in this study.

**Figure 7.**
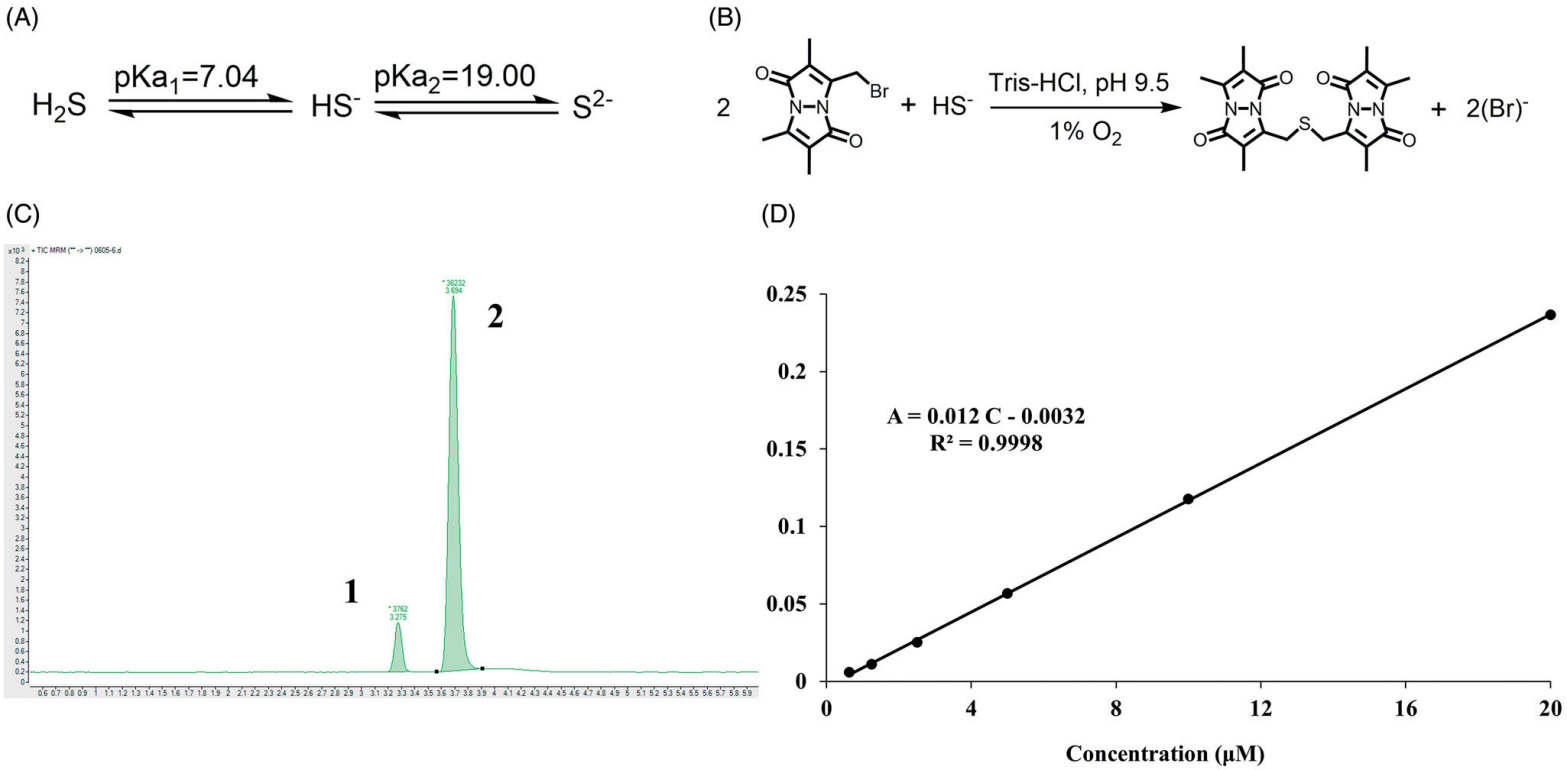
The detection of endogenous H_2_S via LC-MS. (A) The acid dissociation constant of H_2_S and (B) the mechanism of MBB react with HS^−^ produce SDB at alkaline and hypoxia environment; (C) The chromatography of SDB, peak 1: SDB, peak 2: hydrocortisone (internal standard); (D) The calibration curve of SDB in different concentration (0.625 μM–20 μM).

The H_2_S exists in forms of hydrogen sulfide (H_2_S), hydrogen sulfide anion (HS^−^), and sulfide anion (S^2−^) (Figure 7(A)). However, the H_2_S/HS^−^/S^2−^ might react quickly with MBB to produce SDB (Figure 7(B)**)**, and meantime, SDB could remain stable approximately for a period both *in vitro* and *in vivo* (Shen et al. 2011; Shen, Chakraborty, et al. 2014). With LC-MS, two peaks were detected, while SDB was peak 1 and internal standard was peak 2 (Figure 7(C)). To calculate the real-time H_2_S concentration both *in vitro* and *in vivo*, the endogenous H_2_S concentration was calculated according to the calibration curve of SDB in the range of concentration from 0.625 μM to 20 μM (Figure 7(D)).

As reported, the released SPRC would elevate H_2_S levels via the CSE/H_2_S pathway *in vivo*, (MA et al. 2011; Zheng et al. 2011) and this pathway was also verified again in our study. The short half-life(Ma et al. 2015) of SPRC could result in the excretion before fully reacted with CSE, which lead to an incomplete H_2_S release *in vivo*. However, a longer and higher release of SPRC from SPRC@DMSN could be observed compared with SPRC powder *in vivo* (Figure 4). Prolonged release of H_2_S was mainly because of the sustained release of SPRC from SPRC@DMSN. Besides, the central radially structure of pore channel enabled the perfusion of liquid medium, leading an accelerating degradation and higher biocompatibility of DMSN (Shen, Yang, *et al.* 2014).

Our group previously reported that daily administration of SPRC at dose of 100 mg kg^−1^ could alleviate the AIA symptoms in rats (Wu et al. 2016). However, in this study, the SPRC group showed low therapeutical effect (Figure 5), which indicated an important role of maintaining H_2_S plasma concentration for the treatment of AIA. The SPRC@DMSN could elevate the H_2_S concentration for 3 days with single oral administration, while the SPRC lasted for only 6 h (Figure 4).

Since bone erosion is also a key index for evaluation of AIA symptoms, the 3 D micro-CT was employed the effect on inhibition of bone erosion in AIA model after treatment (Figure 5H). The AIA model groups showed the severest bone erosion while the SPRC@DMSN-treated group significantly alleviate the symptom, successfully suggesting prevention of bone damage, and this therapeutic effect was potentially through the elevation of endogenous H_2_S.

As previous reported, (Ma et al. 2019; Alaaeldin et al. 2021) the decrease of body weight could be a sign of the establishment of AIA. At the 30th day, the body weight of control group was the highest among all groups, while AIA group showed the lowest body weight. At meantime, SPRC group showed the similar body weight change potentially due to its low therapeutic effect. However, the body weight of SPRC@DMSN was different with the AIA group, which potentially indicated its high therapeutic effect toward AIA in rats **(**Figure 6(A)**)**.

## Conclusions

In this study, DMSN was successfully prepared, and afterwards, the SPRC was successfully loaded into DMSN, which could sustain release SPRC, hence, attaining the sustained releasing manner of H_2_S both *in vitro* and *in vivo*. Potentially owing to sustained release of SPRC from SPRC@DMSN, the SPRC@DMSN not only showed a longer H_2_S release *in vivo*, but also a better therapeutic effect for AIA in rats. Taken together, DMSN could be a novel favorable drug carrier with low toxicity and high biocompatibility for the treatment of RA.
